# Supplementary material for: Interspecific Hybridization and Complete Mitochondrial Genome Analysis of Two Ghost Moth Species
Source: Insects. 2021 Nov 21;12(11):1046. doi: 10.3390/insects12111046 (PMC8625261; doi:10.3390/insects12111046)
Supplement: Supplementary file 1 [file insects-12-01046-s001.zip › Supplementary Figure S1. Comparison of the fresh weights of the larvae of inbred and hybrid populations during the culture times.pdf]

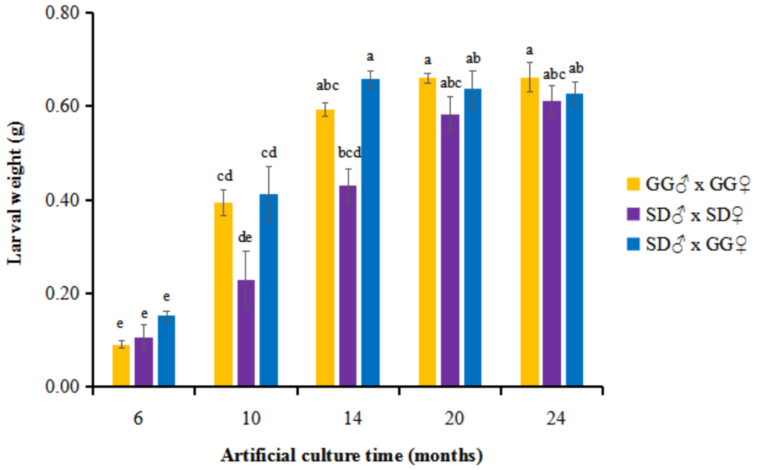

Supplementary Figure S1. Comparison of the fresh weights of the larvae of inbred and hybrid populations during the culture times. The columns with same letters indicated no significant differences ($p>0.05$).
