# Supplementary material for: Interspecific Hybridization and Complete Mitochondrial Genome Analysis of Two Ghost Moth Species
Source: Insects. 2021 Nov 21;12(11):1046. doi: 10.3390/insects12111046 (PMC8625261; doi:10.3390/insects12111046)
Supplement: Supplementary file 1 [file insects-12-01046-s001.zip › Supplementary Figure S3. Secondary structures of 22 tRNAs encoded by the Thitarodes mitochondrial genomes.pdf]

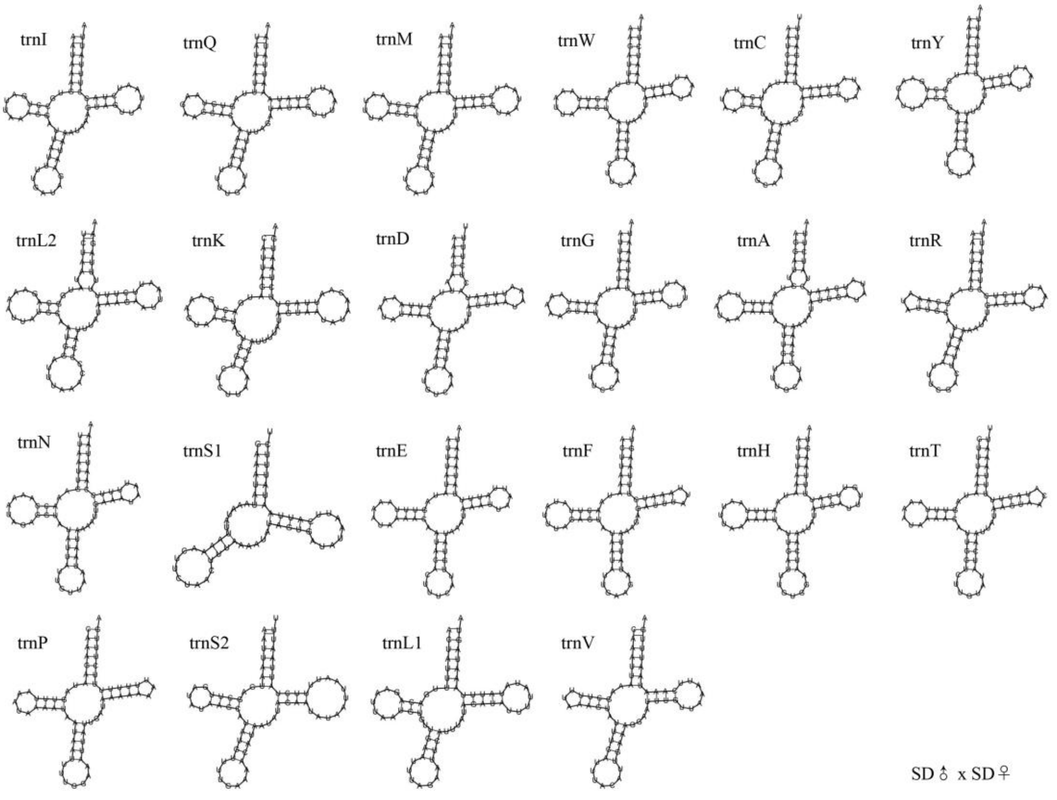
trnI
trnQ
trnM
trnW
trnC
trnY
trnL2
trnK
trnD
trnG
trnA
trnR
trnN
trnS1
trnE
trnF
trnH
trnT
trnP
trnS2
trnL1
trnV
SD♂ x SD♀

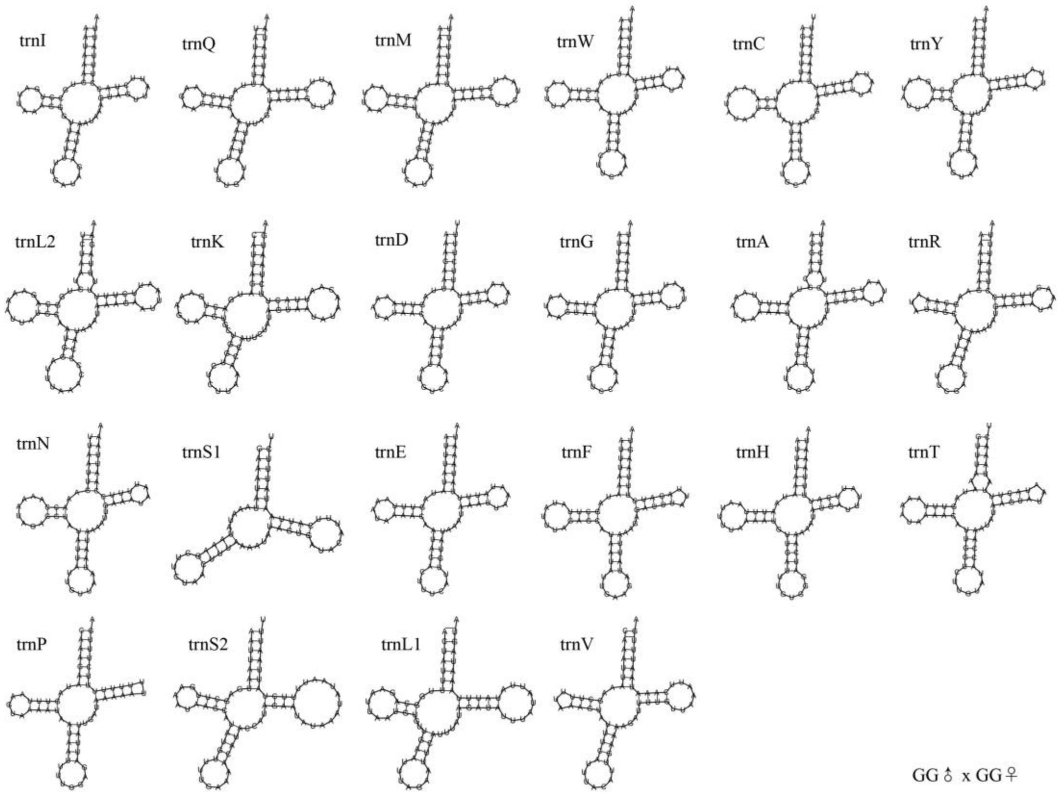
trnI
trnQ
trnM
trnW
trnC
trnY
trnL2
trnK
trnD
trnG
trnA
trnR
trnN
trnS1
trnE
trnF
trnH
trnT
trnP
trnS2
trnL1
trnV
GG♂ x GG♀

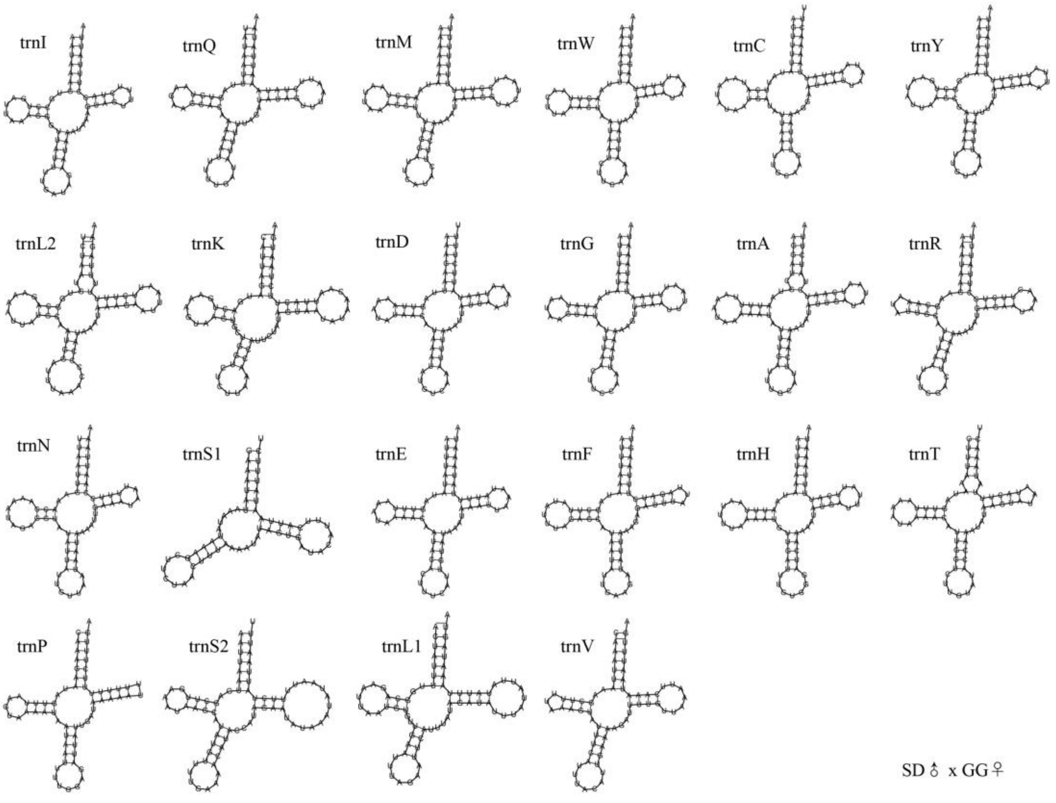

Supplementary Figure S3. Secondary structures of 22 tRNAs encoded by the *Thitarodes* mitochondrial genomes. The tRNAs are labeled with the abbreviations for their corresponding amino acids.
