# Supplementary material for: Interspecific Hybridization and Complete Mitochondrial Genome Analysis of Two Ghost Moth Species
Source: Insects. 2021 Nov 21;12(11):1046. doi: 10.3390/insects12111046 (PMC8625261; doi:10.3390/insects12111046)
Supplement: Supplementary file 1 [file insects-12-01046-s001.zip › Supplementary Figure S4. Alignment of overlapping region between atp8 and atp6 across three Thitarodes populations.pdf]

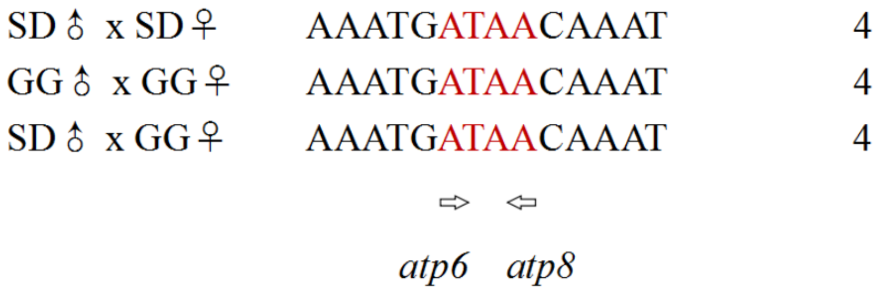

Supplementary Figure S4. Alignment of overlapping region between *atp8* and *atp6* across three *Thitarodes* populations
