# Supplementary material for: Interspecific Hybridization and Complete Mitochondrial Genome Analysis of Two Ghost Moth Species
Source: Insects. 2021 Nov 21;12(11):1046. doi: 10.3390/insects12111046 (PMC8625261; doi:10.3390/insects12111046)
Supplement: Supplementary file 1 [file insects-12-01046-s001.zip › Supplementary Figure S5. The phylogenetic relationship constructed by the amino acid sequence.pdf]

(a) *atp6*

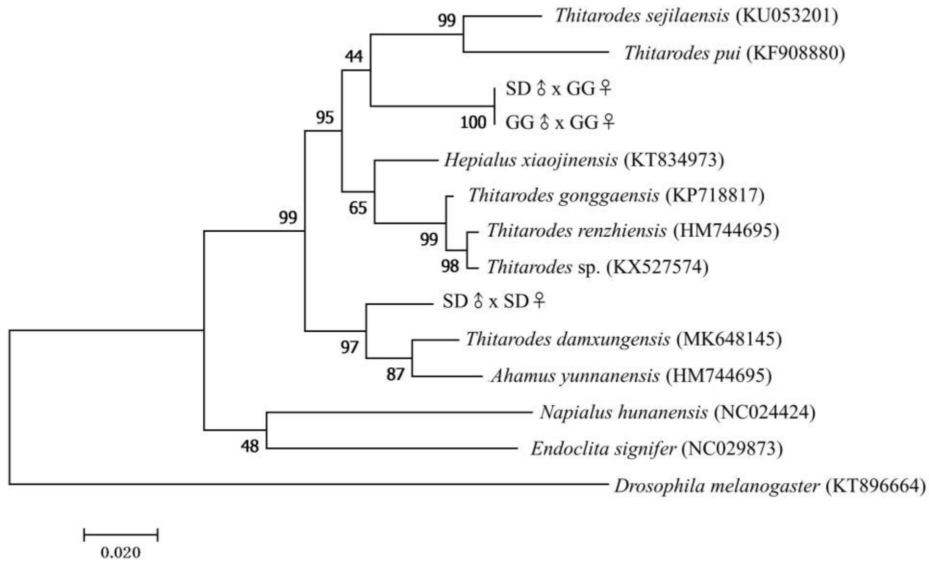

99
Thitarodes sejilaensis (KU053201)
Thitarodes pui (KF908880)
44
SD ♂ x GG ♀
95
100
GG ♂ x GG ♀
Hepialus xiaojinensis (KT834973)
Thitarodes gonggaensis (KP718817)
65
99
Thitarodes renzhiensis (HM744695)
99
98
Thitarodes sp. (KX527574)
SD ♂ x SD ♀
97
Thitarodes damxungensis (MK648145)
87
Ahamus yunnanensis (HM744695)
Napialus hunanensis (NC024424)
48
Endoclita signifer (NC029873)
Drosophila melanogaster (KT896664)
0.020

(b) *atp8*

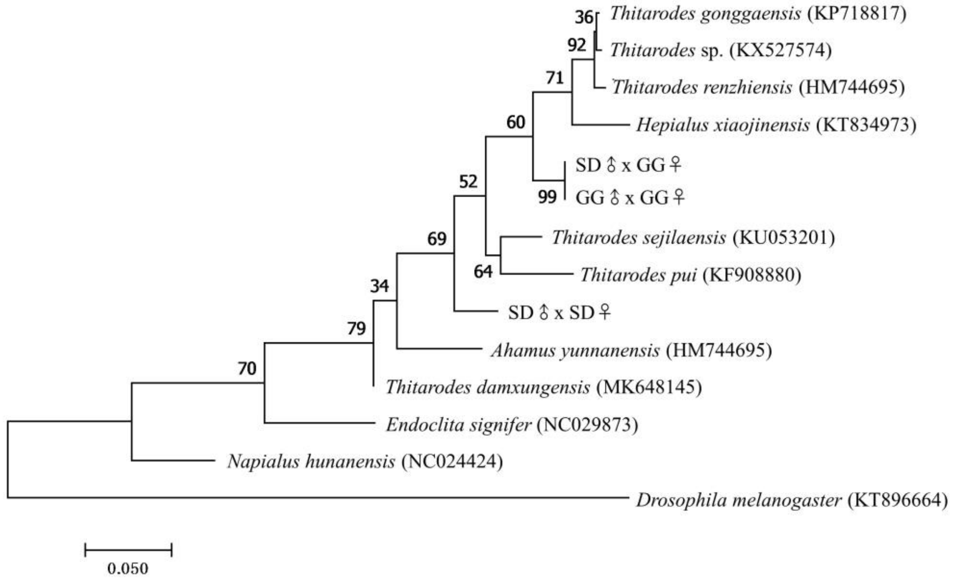

36
Thitarodes gonggaensis (KP718817)
92
Thitarodes sp. (KX527574)
71
Thitarodes renzhiensis (HM744695)
60
Hepialus xiaojinensis (KT834973)
SD ♂ x GG ♀
52
99
GG ♂ x GG ♀
69
Thitarodes sejilaensis (KU053201)
64
Thitarodes pui (KF908880)
34
SD ♂ x SD ♀
79
Ahamus yunnanensis (HM744695)
70
Thitarodes damxungensis (MK648145)
Endoclita signifer (NC029873)
Napialus hunanensis (NC024424)
Drosophila melanogaster (KT896664)
0.050

(c) *cytb*

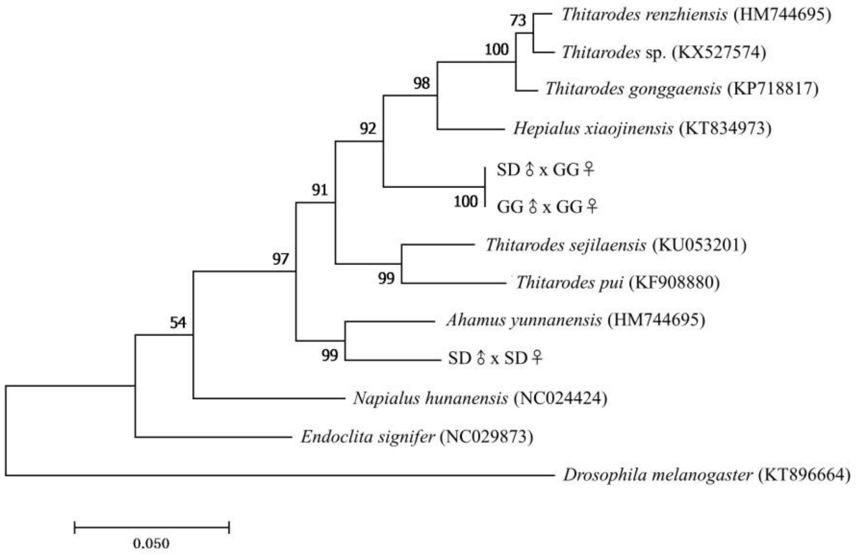
Thitarodes renzhiensis (HM744695)
Thitarodes sp. (KX527574)
Thitarodes gonggaensis (KP718817)
Hepialus xiaojinensis (KT834973)
SD ♂ x GG ♀
GG ♂ x GG ♀
Thitarodes sejilaensis (KU053201)
Thitarodes pui (KF908880)
Ahamus yunnanensis (HM744695)
SD ♂ x SD ♀
Napialus hunanensis (NC024424)
Endoclita signifer (NC029873)
Drosophila melanogaster (KT896664)
73
100
98
92
100
91
97
99
54
99
0.050

(d) *cox1*

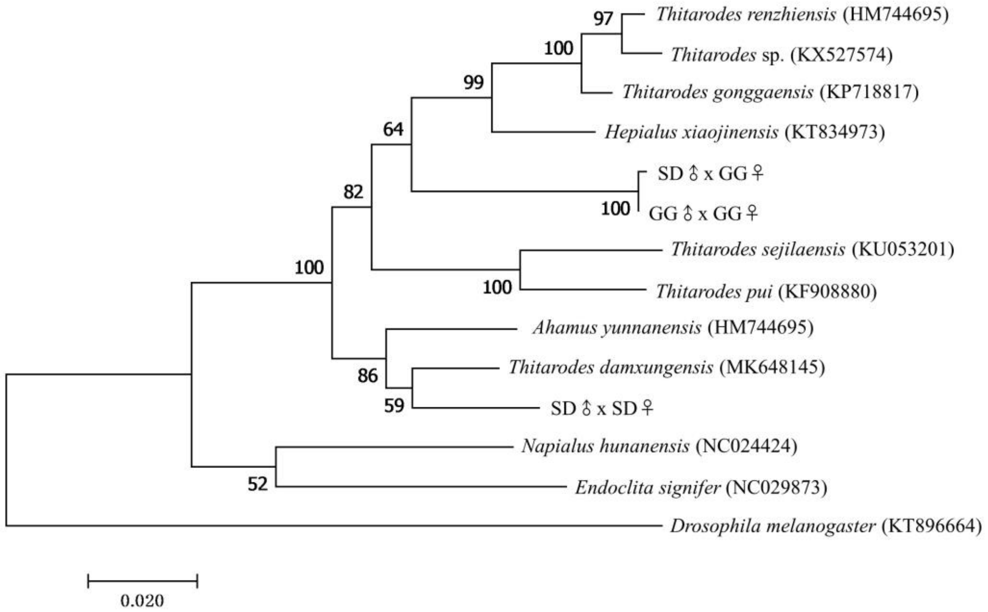
Thitarodes renzhiensis (HM744695)
Thitarodes sp. (KX527574)
Thitarodes gonggaensis (KP718817)
Hepialus xiaojinensis (KT834973)
SD ♂ x GG ♀
GG ♂ x GG ♀
Thitarodes sejilaensis (KU053201)
Thitarodes pui (KF908880)
Ahamus yunnanensis (HM744695)
Thitarodes damxungensis (MK648145)
SD ♂ x SD ♀
Napialus hunanensis (NC024424)
Endoclita signifer (NC029873)
Drosophila melanogaster (KT896664)
97
100
99
64
100
82
100
100
86
59
52
0.020

(e) c*ox2*

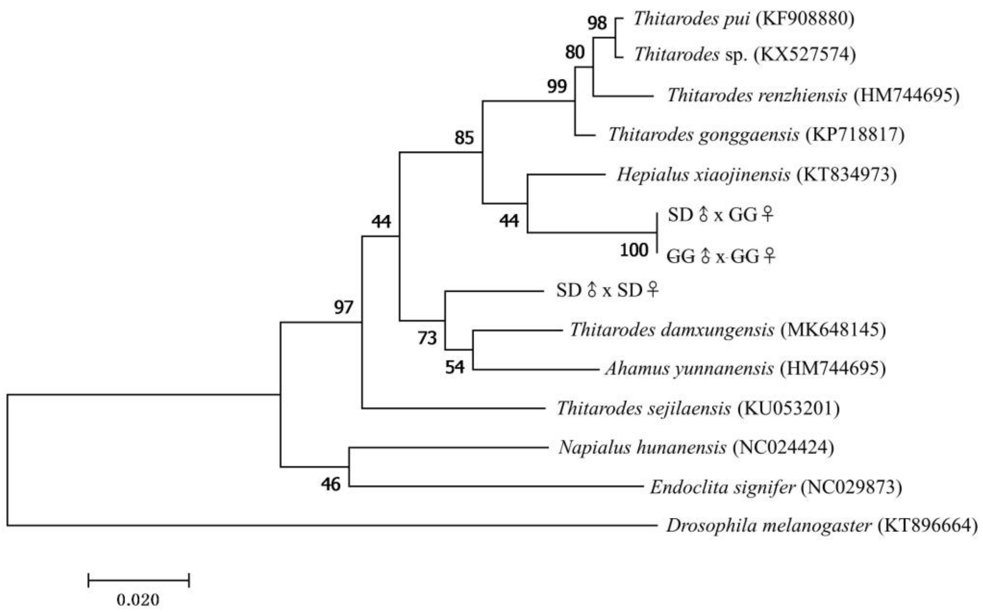

98
Thitarodes pui (KF908880)
80
Thitarodes sp. (KX527574)
99
Thitarodes renzhiensis (HM744695)
Thitarodes gonggaensis (KP718817)
85
Hepialus xiaojinensis (KT834973)
44
SD ♂ x GG ♀
44
100
GG ♂ x GG ♀
SD ♂ x SD ♀
97
Thitarodes damxungensis (MK648145)
73
54
Ahamus yunnanensis (HM744695)
Thitarodes sejilaensis (KU053201)
Napialus hunanensis (NC024424)
46
Endoclita signifer (NC029873)
Drosophila melanogaster (KT896664)
0.020

(f) c*ox3*

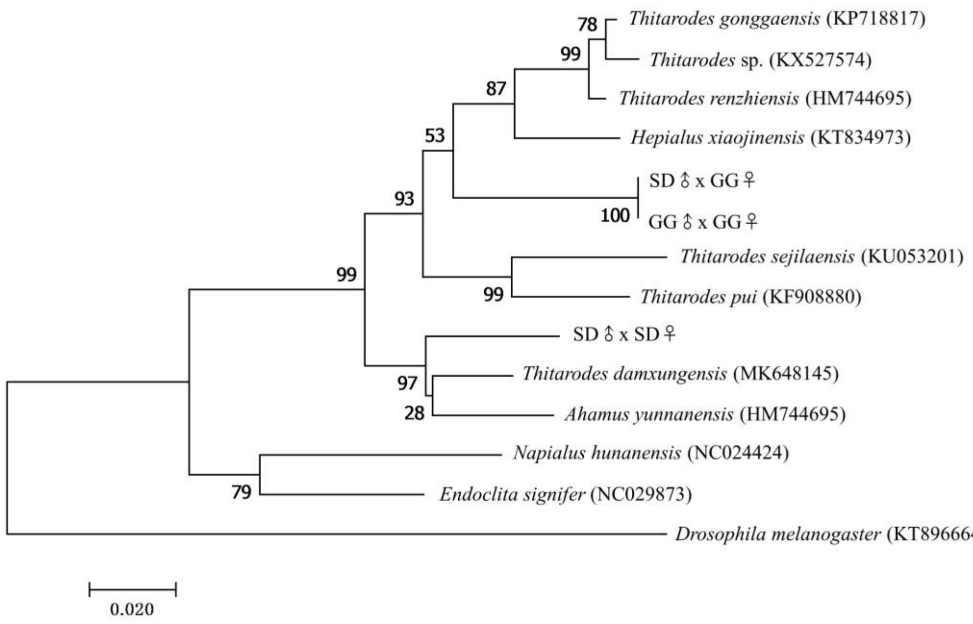

78
Thitarodes gonggaensis (KP718817)
99
Thitarodes sp. (KX527574)
87
Thitarodes renzhiensis (HM744695)
53
Hepialus xiaojinensis (KT834973)
SD ♂ x GG ♀
93
100
GG ♂ x GG ♀
Thitarodes sejilaensis (KU053201)
99
99
Thitarodes pui (KF908880)
SD ♂ x SD ♀
Thitarodes damxungensis (MK648145)
97
28
Ahamus yunnanensis (HM744695)
Napialus hunanensis (NC024424)
79
Endoclita signifer (NC029873)
Drosophila melanogaster (KT896664)
0.020

(g) *nad1*

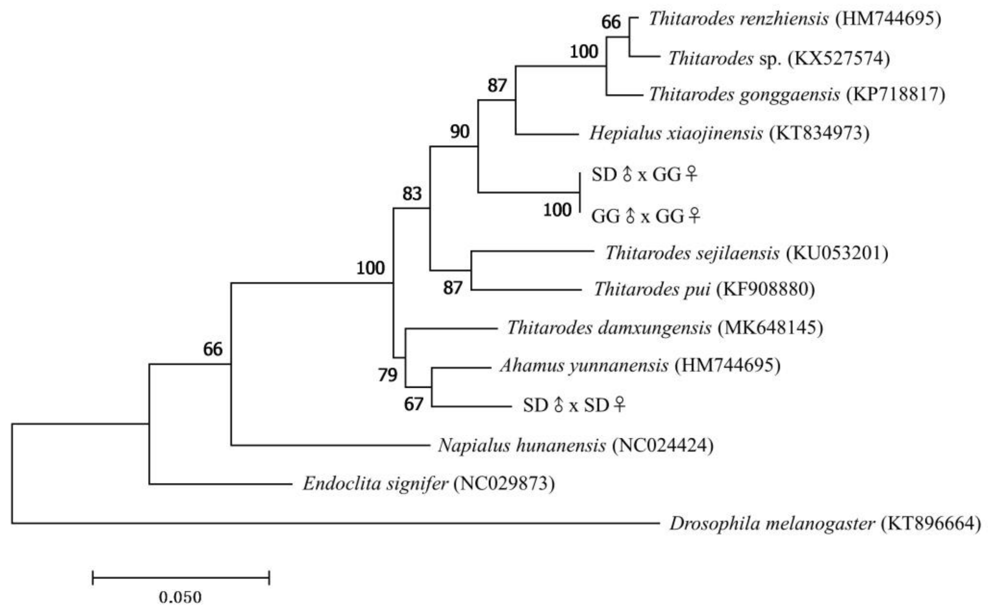

66
Thitarodes renzhiensis (HM744695)
100
Thitarodes sp. (KX527574)
87
Thitarodes gonggaensis (KP718817)
90
Hepialus xiaojinensis (KT834973)
SD ♂ x GG ♀
83
100
GG ♂ x GG ♀
Thitarodes sejilaensis (KU053201)
100
87
Thitarodes pui (KF908880)
Thitarodes damxungensis (MK648145)
66
Ahamus yunnanensis (HM744695)
79
67
SD ♂ x SD ♀
Napialus hunanensis (NC024424)
Endoclita signifer (NC029873)
Drosophila melanogaster (KT896664)
0.050

(h) *nad2*

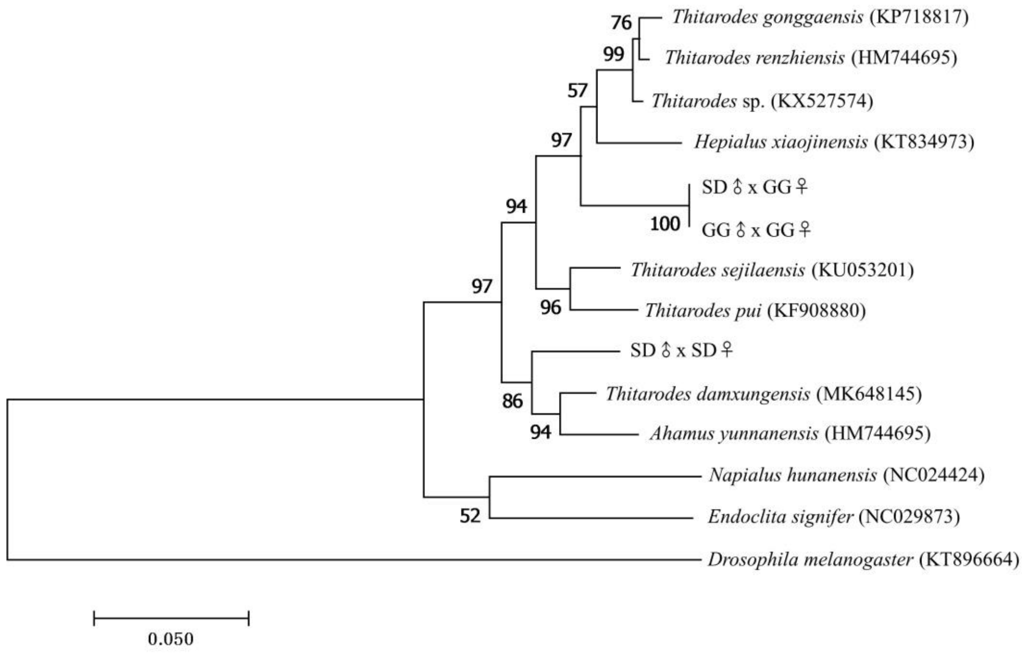

76
Thitarodes gonggaensis (KP718817)
99
Thitarodes renzhiensis (HM744695)
57
Thitarodes sp. (KX527574)
97
Hepialus xiaojinensis (KT834973)
SD ♂ x GG ♀
94
100
GG ♂ x GG ♀
Thitarodes sejilaensis (KU053201)
97
96
Thitarodes pui (KF908880)
SD ♂ x SD ♀
Thitarodes damxungensis (MK648145)
86
94
Ahamus yunnanensis (HM744695)
Napialus hunanensis (NC024424)
52
Endoclita signifer (NC029873)
Drosophila melanogaster (KT896664)
0.050

(i) ***nad3***

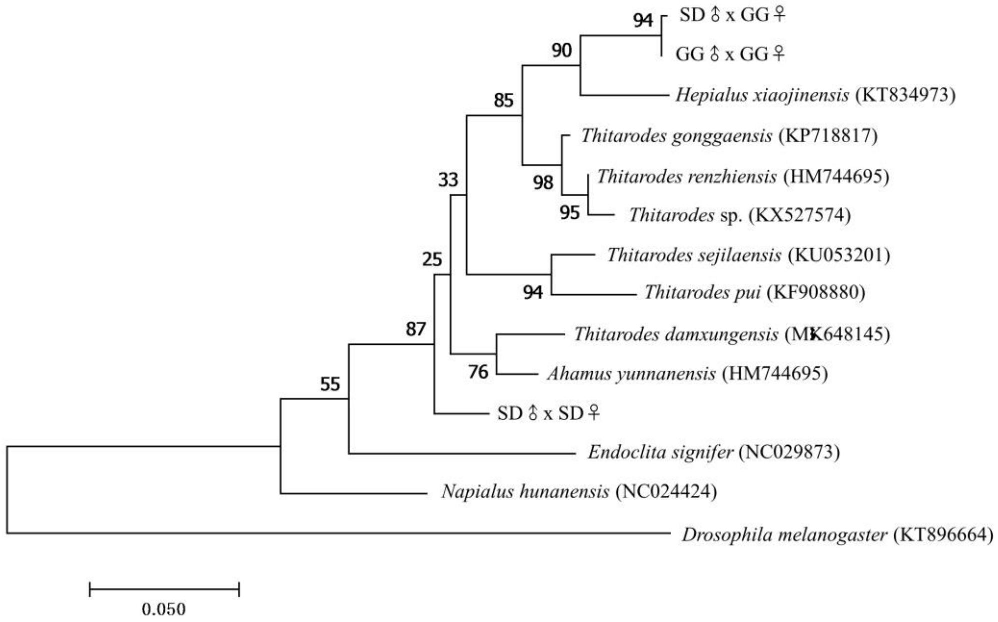
SD ♂ x GG ♀
GG ♂ x GG ♀
Hepialus xiaojinensis (KT834973)
Thitarodes gonggaensis (KP718817)
Thitarodes renzhiensis (HM744695)
Thitarodes sp. (KX527574)
Thitarodes sejilaensis (KU053201)
Thitarodes pui (KF908880)
Thitarodes damxungensis (MK648145)
Ahamus yunnanensis (HM744695)
SD ♂ x SD ♀
Endoclita signifer (NC029873)
Napialus hunanensis (NC024424)
Drosophila melanogaster (KT896664)
94
90
85
98
95
33
94
25
87
76
55
0.050

(j) ***nad 4***

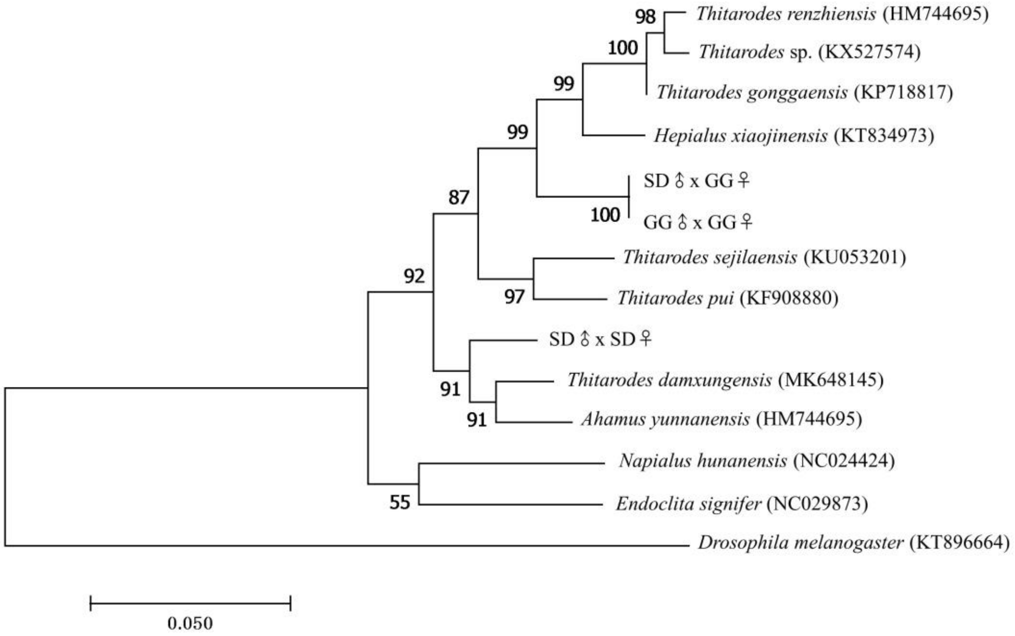
Thitarodes renzhiensis (HM744695)
Thitarodes sp. (KX527574)
Thitarodes gonggaensis (KP718817)
Hepialus xiaojinensis (KT834973)
SD ♂ x GG ♀
GG ♂ x GG ♀
Thitarodes sejilaensis (KU053201)
Thitarodes pui (KF908880)
SD ♂ x SD ♀
Thitarodes damxungensis (MK648145)
Ahamus yunnanensis (HM744695)
Napialus hunanensis (NC024424)
Endoclita signifer (NC029873)
Drosophila melanogaster (KT896664)
98
100
99
99
87
100
92
97
91
91
55
0.050

(k) *nad4L*

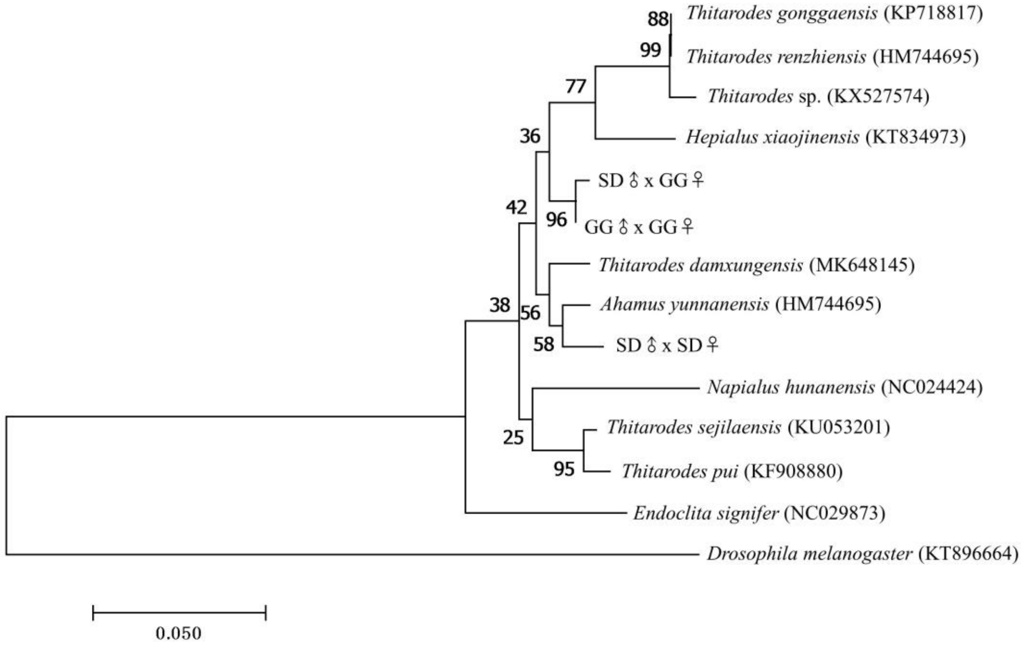

(l) *nad5*

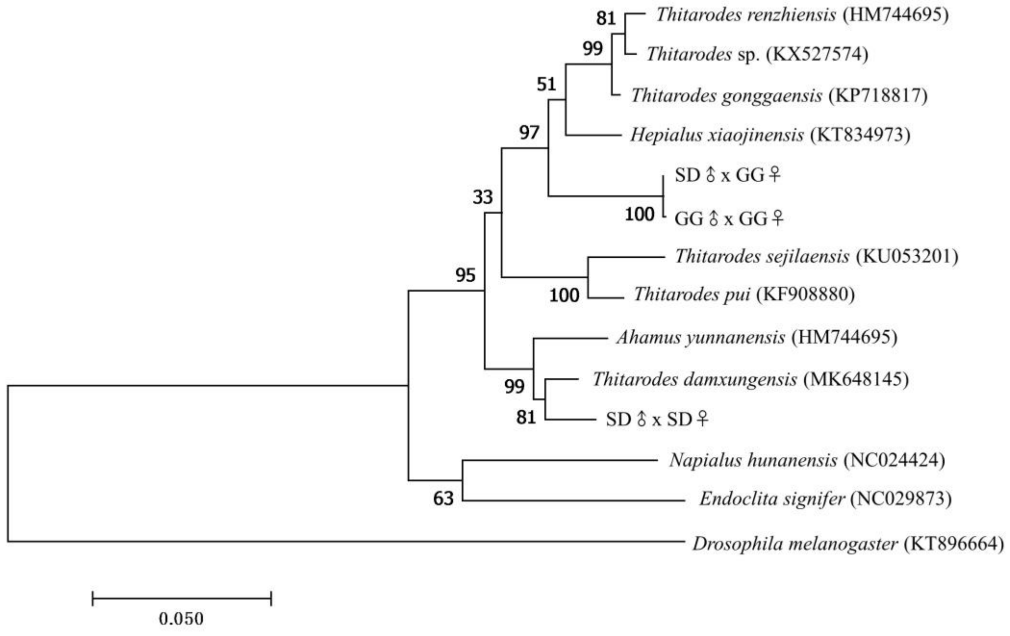

(m) *nad6*

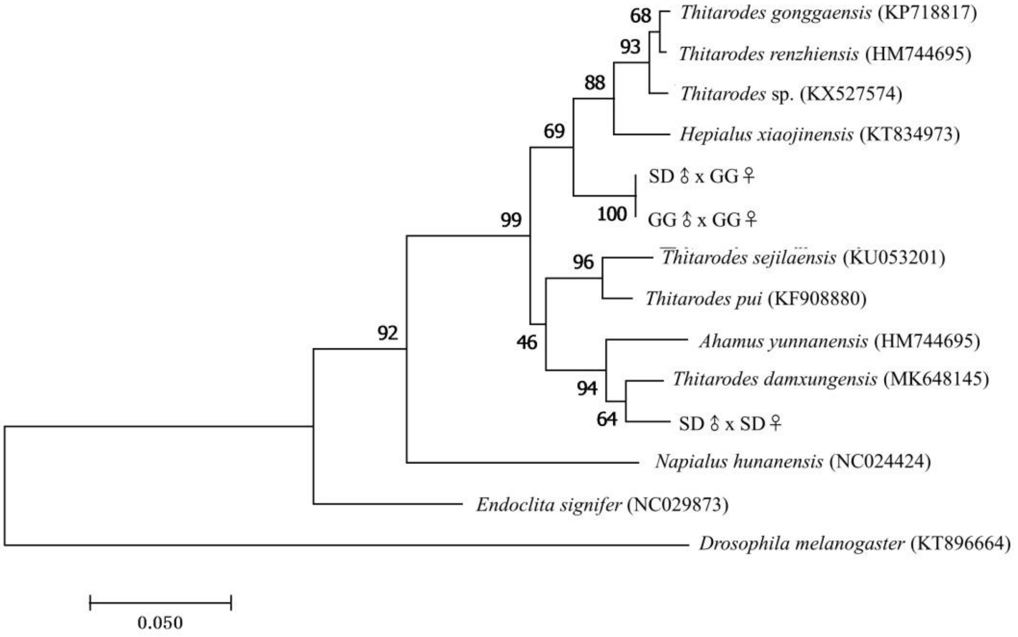

Supplementary Figure S5. The phylogenetic relationship constructed by the amino acid sequence derived from each PCG among 13 mitogenomes of Hepialidae insects and 1 outgroup.
