# Supplementary material for: Interspecific Hybridization and Complete Mitochondrial Genome Analysis of Two Ghost Moth Species
Source: Insects. 2021 Nov 21;12(11):1046. doi: 10.3390/insects12111046 (PMC8625261; doi:10.3390/insects12111046)
Supplement: Supplementary file 1 [file insects-12-01046-s001.zip › Supplementary Table S1. Egg characters from the inbred and hybrid populations.pdf]

Supplementary Table S1. Egg characters from the inbred and hybrid populations

Combinations	Eggs in one milliliter	The weight of one thousand eggs (g)	Eggs per female	Hatching rates (%)
SD♂ x SD♀	3976±109 a	0.18±0.01 c	512±3 a	24.16±1.76 a
GG♂ x GG♀	2955±69 b	0.31±0.02 a	411±20 b	26.64±2.42 a
SD♂ x GG♀	2800±73 b	0.28±0.02 ab	347±8 b	12.62±2.80 b

Note: the columns with the same letters indicated no significant differences (p>0.05).
