# Supplementary material for: Interspecific Hybridization and Complete Mitochondrial Genome Analysis of Two Ghost Moth Species
Source: Insects. 2021 Nov 21;12(11):1046. doi: 10.3390/insects12111046 (PMC8625261; doi:10.3390/insects12111046)
Supplement: Supplementary file 1 [file insects-12-01046-s001.zip › Supplementary Table S2. Mummification rates of the inbred and hybrid Thitarodes larvae challenged with O. sinensis fungus.pdf]

Supplementary Table S2. Mummification rates of the inbred larvae at 120 days post infection with *O. sinensis* fungus

Populations	Fungus isolates			
	**KD**	**XZ**	**QH**	**YN**
SD♂ x SD♀	55.56±3.57% Aa	32.96±1.48% Ab	62.59±3.76% Ba	53.89±0.56% Aa
GG♂ x GG♀	49.63±2.43% Aa	20.37±5.38% Ab	62.96±1.96% Ba	51.48±2.96% Aa

Note: The columns with different capital letters indicated significant differences ($p>0.05$). The lines with different lowercase letters indicated significant differences ($p>0.05$).
