# Supplementary material for: Interspecific Hybridization and Complete Mitochondrial Genome Analysis of Two Ghost Moth Species
Source: Insects. 2021 Nov 21;12(11):1046. doi: 10.3390/insects12111046 (PMC8625261; doi:10.3390/insects12111046)
Supplement: Supplementary file 1 [file insects-12-01046-s001.zip › Supplementary Table S3. Fresh weight of infected and uninfected larvae at 120 days post infection with different O. sinensis fungal strains.pdf]

Supplementary Table S3. Fresh weight of infected and uninfected larvae at 120 days post infection with different *O. sinensis* fungal strains

**Fungal Strains**	**GG♂ x GG♀**	**SD♂ x SD♀**
KD	0.67±0.04 A	0.53±0.04 a
XZ	0.67±0.05 A	0.53±0.04 a
QH	0.65±0.05 A	0.53±0.03 a
YN	0.58±0.03 A	0.54±0.04 a
PBS (Solvent control)	0.60±0.03 A	0.56±0.04 a
CK (Uninfected control)	0.59±0.02 A	0.47±0.02 a

Note：Means (±SE) within a column followed by the same letter are not significantly different ($p>0.05$).
